# Adenovirus delivery of encoded monoclonal antibody protects against different types of influenza virus infection

**DOI:** 10.1038/s41541-020-0206-5

**Published:** 2020-07-09

**Authors:** Xiang Wang, Ping Zhou, Mangteng Wu, Kaiyan Yang, Jingao Guo, Xuchen Wang, Jun Li, Zihao Fang, Guiqin Wang, Man Xing, Dongming Zhou

**Affiliations:** 1 Shanghai Public Health Clinical Center, Fudan University, Shanghai, 201508 China; 2 Vaccine Research Center, CAS Key Laboratory of Molecular Virology and Immunology, Institut Pasteur of Shanghai, Chinese Academy of Sciences, Shanghai, 200031 China; 3 University of Chinese Academy of Sciences, Beijing, 100049 China; 4 Department of Pathogen Biology, School of Basic Medical Sciences, Tianjin Medical University, Tianjin, 300070 China

**Keywords:** Biotechnology, Immunology, Microbiology

## Abstract

Due to the high mutation and recombination rates of the influenza virus, current clinically licensed influenza vaccines and anti-influenza drugs provide limited protection against the emerging influenza virus epidemic. Therefore, universal influenza vaccines with high efficacy are urgently needed to ensure human safety and health. Passive immunization of influenza broadly neutralizing antibodies may become an ideal option for controlling influenza infection. CR9114 isolated from the peripheral blood mononuclear cells of healthy donors is a broadly neutralizing monoclonal antibody that targets different types of influenza viruses. As the adenovirus vector is one of the most promising delivery vehicles, we employed the chimpanzee adenoviral vector, AdC68, to express CR9114 as a universal anti-influenza vaccine, termed AdC68-CR9114, and evaluated its antibody expression and its broad spectrum of prophylactic and therapeutic effects in animal models. Based on our findings, AdC68-CR9114-infected cell expressed the broadly neutralizing antibody at a high level in vitro and in vivo, exhibited biological functions, and protected mice from different types of influenza virus infection at different time points. The findings from this study shed light on a new strategy for controlling and preventing influenza infection.

## Introduction

The influenza virus continues to serve as a severe threat to human health. It causes serious disease and even death among susceptible populations, as well as direct and indirect economic losses. According to the World Health Organization (WHO), seasonal influenza virus causes approximately 5 million infections and 250,000–500,000 deaths worldwide every year^1^. The WHO published the 2019–2030 Global Influenza Strategy (GIS)^2^ with the objectives of reducing the burden of seasonal influenza, minimizing the risk of zoonotic influenza, and preparing for the next influenza pandemic in all countries. The GIS has stressed the importance of influenza vaccines, drugs, and their shortages, thereby highlighting the urgent need to diversify and optimize current production capabilities and technologies, and seek new vaccines that offer stronger, broader, more long-lasting protection, and can be more rapidly produced.

Traditional influenza vaccines have been well-studied. Further, they have had a long history of use; however, their efficacy is poor in individuals older than 65 years. The effectiveness of the influenza vaccine is approximately 52% during the seasons when the vaccine and circulating viruses are well-matched^3^. If a significant mutation occurs in the circulating influenza virus, the efficacy of the vaccine is reduced to 36% (https://www.cdc.gov/flu/vaccines-work/effectivenessqa.htm). In addition, traditional vaccine cannot protect humans from a novel outbreak of the influenza virus, such as the H7N9 that occurred in 2013^4^.

With the progress of the monoclonal antibody (mAb) approach, an increasing number of antibodies has been passively used in the prevention and control of infectious diseases^5^. For example, the delivery of mAbs with potent neutralizing activity against a broad range of isolates is a promising approach for the prevention and treatment of HIV infection^6^. The passive transfer of the anti-HIV antibody to a patient was found to significantly reduce the HIV titer^7^. Palivizumab, a newly approved anti-RSV mAb, has been routinely used as prophylaxis against respiratory syncytial virus^8^. To achieve viral infection control, anti-rabies immunoglobulins are usually administered to patients exposed to the rabies virus^9,10^. However, human immunoglobulins are rare and expensive, while equine rabies-immune globulin continues to induce some side effects^9^. Thus, the development of broadly neutralizing antibodies against the rabies virus may be a superior alternative^11^. Recently, the anti-influenza broadly neutralizing antibodies found in infected humans have provided new insights into universal influenza vaccine development^12^. In fact, researchers have attempted several methods to construct hemagglutinin stalk-based vaccines to activate broadly neutralizing antibodies, with minor effects achieved^13^. However, passive immunization of broadly neutralizing antibodies might be the most effective countermeasure for influenza infection.

C179^14^ is the first isolated influenza broadly neutralizing antibody that can neutralize all H1 and H2 strains. In addition, it can cross-neutralize the H5, H6, and H9 strains^15^. Compared with other broadly neutralizing mAbs that cross-neutralize several different subtypes of influenza A virus, such as CR6261, F10, 12D1, CR8020, and FI6^15^, CR9114^16^ is one of the most broadly neutralizing antibodies that has been identified to date. CR9114 binds to a conserved epitope in the HA stem and protects against lethal challenge from influenza A or B viruses. As a result, some broadly neutralizing antibodies against influenza have been used in clinical trials. The administration of the anti-HA antibody (VIS410)^17^ and anti-M2e antibody (TCN-032)^18^ significantly reduced the influenza virus titer in subjects. VIS410, a broadly neutralizing antibody engineered to bind the influenza A virus, has completed Ph2a studies in experimentally infected subjects^19^. MedImmune’s MEDI8852^20^ and Genentech’s MHAA4549A have progressed to trials for naturally infected subjects^19^. New advances in biotech are also paving the way for more cost-effective immunoprophylaxis using broadly neutralizing antibodies^19^.

Because of the instability and high cost of the generic immunoglobulins, their administration to most patients is impractical. Hence, using a viral vector to express antibodies would be an ideal alternative. Adenoviral vectors have been widely used in vaccine development owing to their broad cell tropism, high gene expression, high immunogenicity, and mature production technology^21^. This commonly used adenoviral vector is human serotype 5 (AdHu5), a common-cold virus circulating in humans with a seropositive rate of 40–60%^22,23^. To circumvent the downside of pre-existing immunity to AdHu5, rare serotypes of human adenovirus or those derived from other species, such as chimpanzee, have been developed and applied in vaccine development. Chimpanzee adenoviruses, which rarely circulate in humans, have been engineered to carry different antigens and demonstrate remarkable safety and immunogenicity in clinical studies^24^. In this study, a chimpanzee-origin adenovirus vector with E1/E3 deletion, AdC68, was selected to express the broadly influenza-neutralizing antibody, CR9114, as a universal influenza vaccine, termed AdC68–CR9114, to offer both prophylactic and therapeutic protection against influenza infection.

## Results

### Expression of CR9114 in vitro and in vivo

**Fig. 1:**
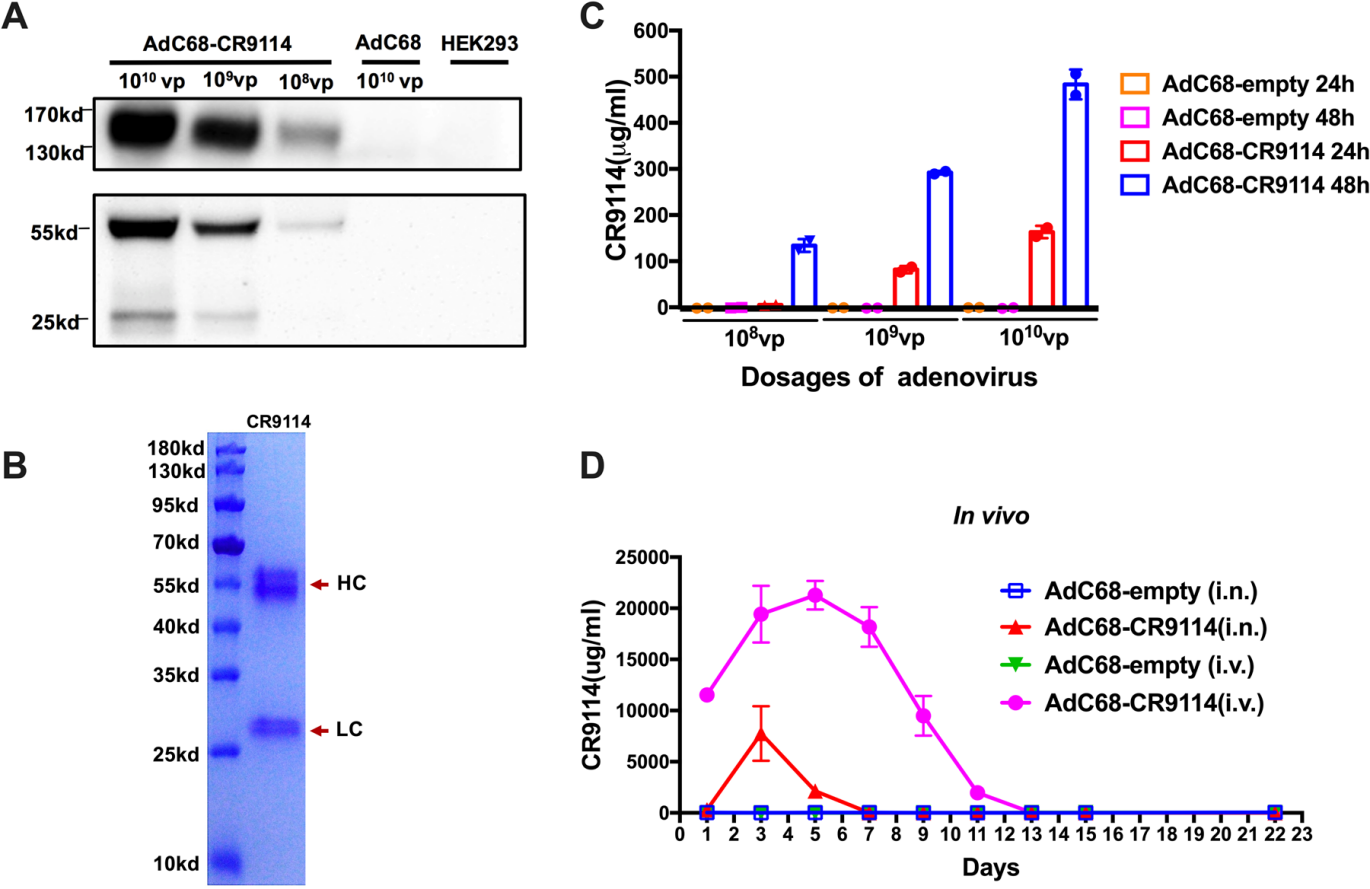
CR9114 expression in vitro and in vivo. To measure the CR9114 antibody level in vitro, different quantities of AdC68–CR9114 (10^10^, 10^9^, or 10^8^ virus particles (vp)) were used to infect the HEK293 cells in a 6-well plate. AdC68-gp (10^10^ vp) was used as the negative control, while the uninfected HEK293 cells were used as the blank controls. Supernatants were harvested at 24 and 48 h after infection. For the in vivo expression of the CR9114 antibody, mice were randomly divided into four groups (5 mice per group), and intranasally immunized with AdC68–CR9114 or AdC68–empty at 5 × 10^10^ vp/mouse in 30 μl of PBS, or intravenously administered AdC68–CR9114 or AdC68–empty at 5 × 10^10^ vp/mouse in 200 μl of PBS. All mice were subjected to bleeding every other day. **a** Western blot detection of the CR9114 antibody in the supernatant of infected HEK293 cells under nonreducing and reducing conditions. **b** The purified CR9114 antibody was boiled and subjected to SDS-PAGE with Coomassie Blue R-250 staining. **c**, **d** The concentrations of CR9114 in vitro and in vivo (five mice per group). Data in **c**, **d** are expressed as mean ± SD.

To assess the expression of CR9114 in vitro, Western blotting was performed. Different quantities of AdC68–CR9114 (10^8^, 10^9^, or 10^10^ virus particles (vp)) were used to infect HEK293 cells in a 6-well plate. AdC68–empty (10^10^ vp) was used as the negative control, while uninfected HEK293 cells were used as the blank controls. After 24 h, the cell supernatant was analyzed by reducing and nonreducing Western blotting with a horseradish peroxidase (HRP)-conjugated anti-human IgG antibody. As shown in Fig. 1a, the full-length antibody, CR9114, appeared at approximately 160 kDa in the nonreducing condition, but was resolved into the heavy chain (55 kD) and light chain (25 kD) under reducing conditions. For further validation, the CR9114 antibody was purified from the supernatant of infected HEK293 cells using a protein A column and subjected to reducing sodium dodecyl sulphate polyacrylamide gel electrophoresis (SDS-PAGE) with Coomassie Blue R-250 staining. The heavy and light chains are clearly presented in Fig. 1b. Sandwich ELISA was also performed to measure the concentration of CR9114 in the supernatant of infected HEK293 cells. In the cell supernatant, the concentration of the CR9114 antibody was up to 163 µg/ml at 24 h after 10^10^ vp AdC68–CR9114 infection. At 48 h, its concentration increased to 483 µg/ml (Fig. 1c). Therefore, AdC68–CR9114-infected cells efficiently expressed the CR9114 antibody in vitro.

The serum samples of C57BL/6 mice immunized intravenously or intranasally with AdC68–CR9114 were collected every other day to determine the expression of CR9114 in vivo. The antibody was approximately 11,535 μg/ml at 24 h after intravenous immunization and increased as time progressed. Further, its concentration peaked on day 5 (21,284.7 µg/ml), and decreased to 8 μg/ml on day 13. Compared with the intravenously immunized group, the level of the CR9114 antibody in the intranasally immunized group was lower in the serum as it could not be detected at 24 h post immunization; however, it peaked on day 3 (7751.7 µg/ml) and declined to undetectable levels on day 7 post infection (Fig. 1d).

### AdC68–CR9114 prophylactically or therapeutically protects mice from pH1N1 and influenza B virus

**Fig. 2:**
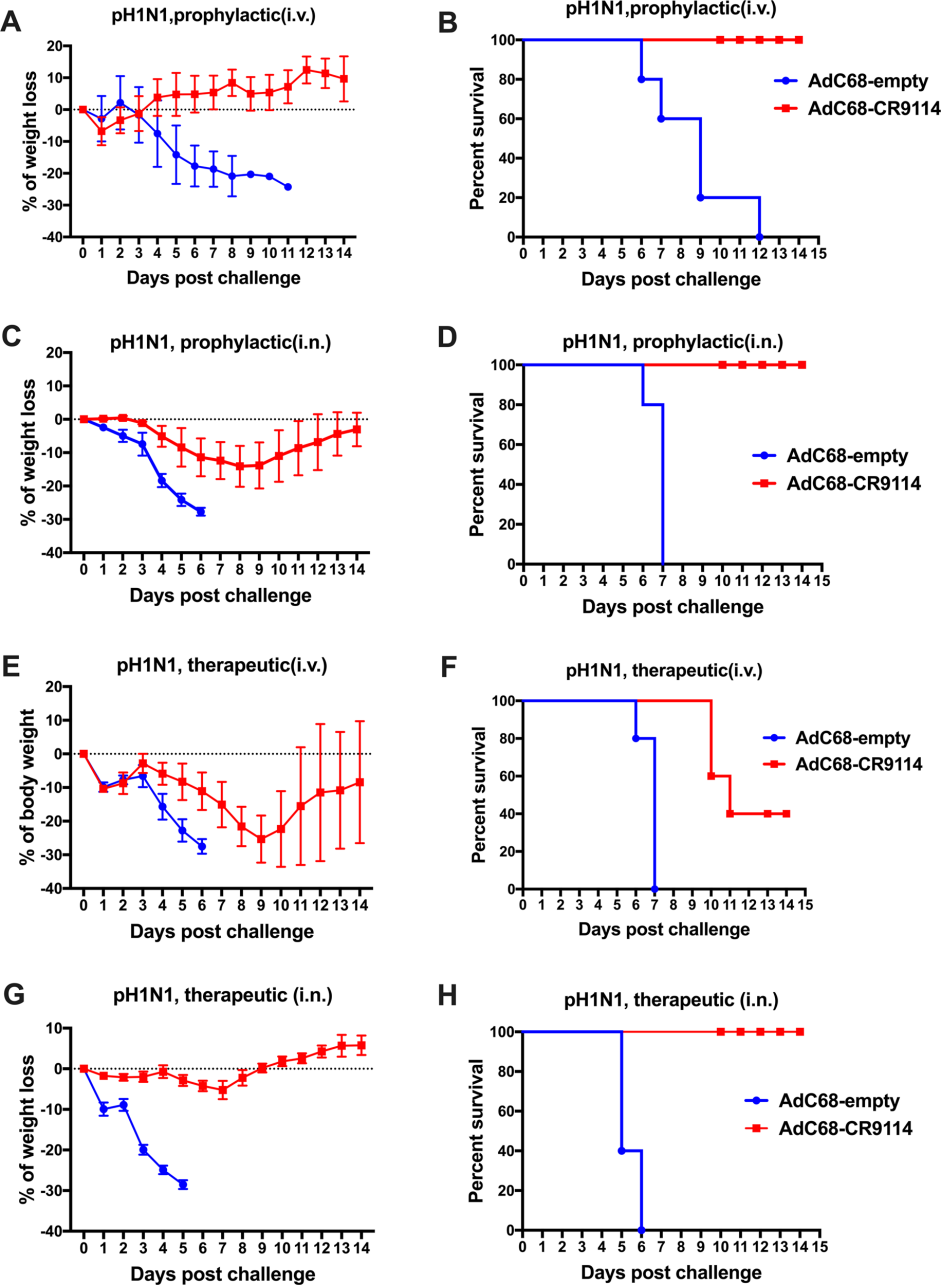
AdC68–CR9114 prophylactically and therapeutically protected mice from pH1N1 infection. For the prophylactic experiments, mice were divided into different groups and immunized with AdC68–CR9114 or AdC68–empty (5 × 10^10^ vp/mouse) via tail intravenous or intranasal immunization. Mice were challenged intranasally with 10LD50 of the pH1N1 influenza virus. Body weight and survival rate were monitored for 2 weeks. For the therapeutic experiments, mice were infected with 10LD50 of the pH1N1 virus, and intravenously or intranasally treated with 5 × 10^10^ vp AdC68–CR9114 or AdC68–empty virus at 6 h following the influenza challenge. **a**, **b** Weight loss and survival rate in the prophylactic group treated intravenously and challenged with pH1N1. **c**, **d** Weight loss and survival rate in the prophylactic group treated intranasally and challenged with pH1N1. **e**, **f** Weight loss and survival rate in the therapeutic groups treated intravenously with AdC68–CR9114 and challenged with pH1N1. **g**, **h** Weight loss and survival rate in the therapeutic group treated intranasally with AdC68–CR9114 and challenged with pH1N1. Five mice in each group. Data in **a**, **c**, **e**, **g** are expressed as mean ± SD.

We used different immunization regimens to determine whether AdC68–CR9114 could prophylactically or therapeutically protect mice from the influenza infection. For the prophylactic experiments, C57BL/6 mice were intravenously administered 5 × 10^10^ vp AdC68–CR9114 virus and challenged with 10LD50 of the pH1N1 (A/California/7/2009) virus after 24 h. In addition, body weight and survival rate were monitored for 14 days. As shown in Fig. 2a, b, mice in the AdC68–CR9114 group rarely experienced any weight loss. In addition, they had a survival rate of 100%. Conversely, weight loss in the AdC68–empty group sharply decreased to 30% on day 11 post challenge; no mice were found to survive. Intranasal immunization was performed to evaluate the prophylactic efficacy of AdC68–CR9114 too. Briefly, C57BL/6 mice were intranasally treated with 5 × 10^10^ vp AdC68–CR9114 virus and challenged with 10LD50 pH1N1 virus after 24 h. As shown in Fig. 2c, d, mice in the control groups died on day 6 post challenge with the pH1N1 virus, whereas mice in the AdC68–CR9114 group showed limited weight loss and recovered with a survival rate of 100%. By comparing the intravenous with the intranasal regimen, the former was identified to be preferred for the prophylactic experiments.

For the therapeutic experiments, mice were first infected with 10LD50 pH1N1 virus, and intravenously or intranasally administered 5 × 10^10^ vp AdC68–CR9114 or AdC68–empty virus 6 h post influenza challenge. As shown in Fig. 2e, f, when intravenously injected, mice in the control group experienced serious weight loss and died on day 6 post challenge, and mice in the AdC68–CR9114 group had a sharp weight loss, with only 40% of mice surviving. In the intranasal immunization experiments, mice in the AdC68–CR9114 group did not experience weight loss and had a survival rate of 100% (Fig. 2g, h). Thus, we selected intranasal treatment for therapeutic use.

**Fig. 3:**
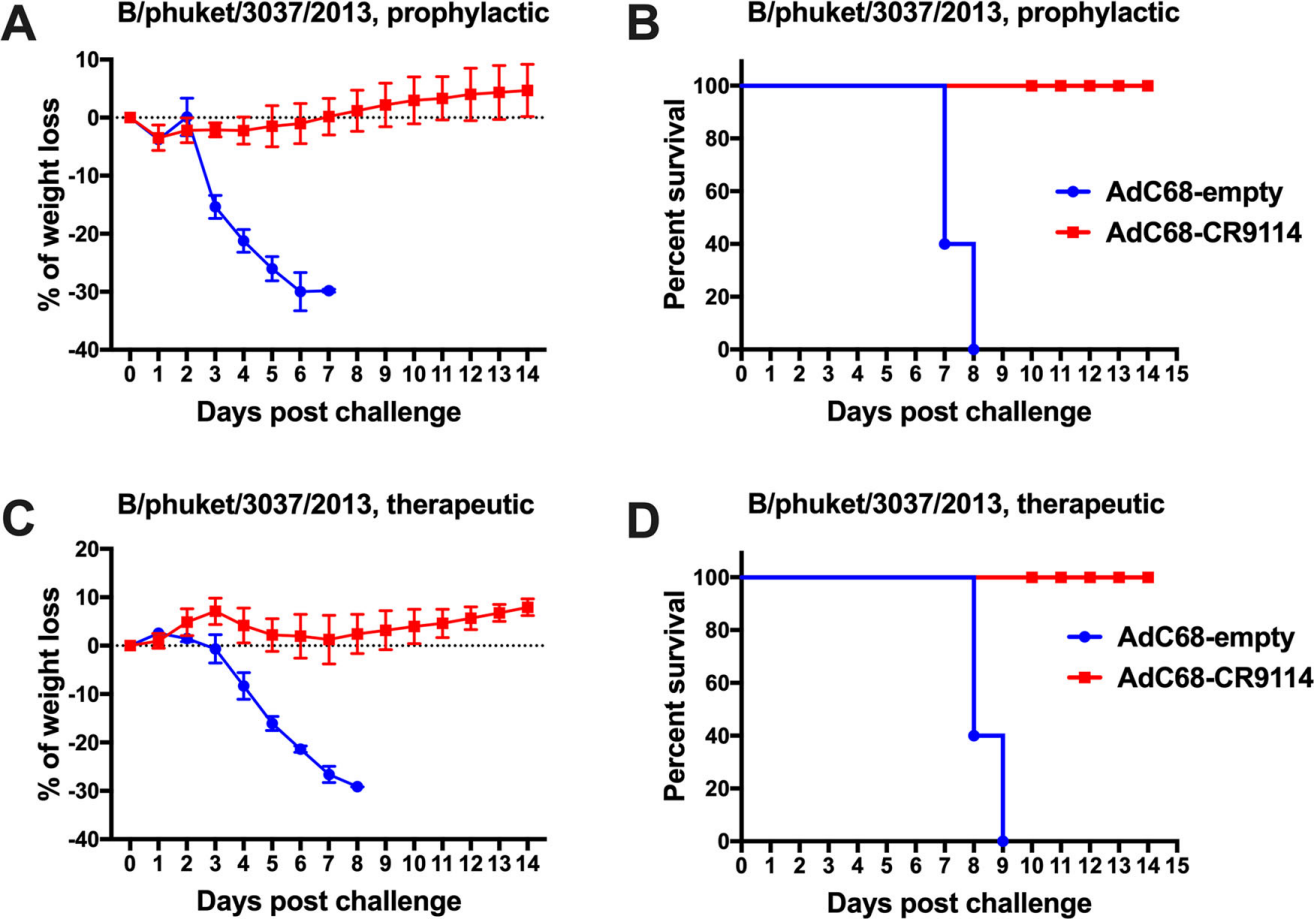
AdC68–CR9114 prophylactically and therapeutically protected mice from influenza B infection. Groups of mice were immunized with AdC68–CR9114 or AdC68–empty (5 × 10^10^ vp/mouse) via tail intravenous (for prophylactic purpose) or intranasal (for therapeutic purpose) immunization. Thereafter, they were challenged intranasally with the influenza B virus. Body weight and survival rate were monitored for 2 weeks. **a**, **b** Weight loss and survival rate in the prophylactic group treated intravenously and challenged with the influenza B virus. **c**, **d** Weight loss and survival rate in the therapeutic group treated intranasally and challenged with the influenza B virus. Five mice in each group. Data in **a**, **c** are expressed as mean ± SD.

The prophylactic and therapeutic protection efficacy of AdC68–CR9114 against influenza B virus was evaluated. As shown in Fig. 3a–d, mice immunized intravenously for prophylactic purpose or treated intranasally with AdC68–CR9114 for therapeutic purpose survived from the influenza B (Yamagata lineage, B/phuket/3037/2013) challenge, whereas those in the control groups showed serious weight loss and mortality. Such findings demonstrate that AdC68–CR9114 protected mice from the influenza A and B infections, prophylactically and therapeutically.

### AdC68–CR9114 prophylactically protects mice from the different subtypes of the influenza virus infection

**Fig. 4:**
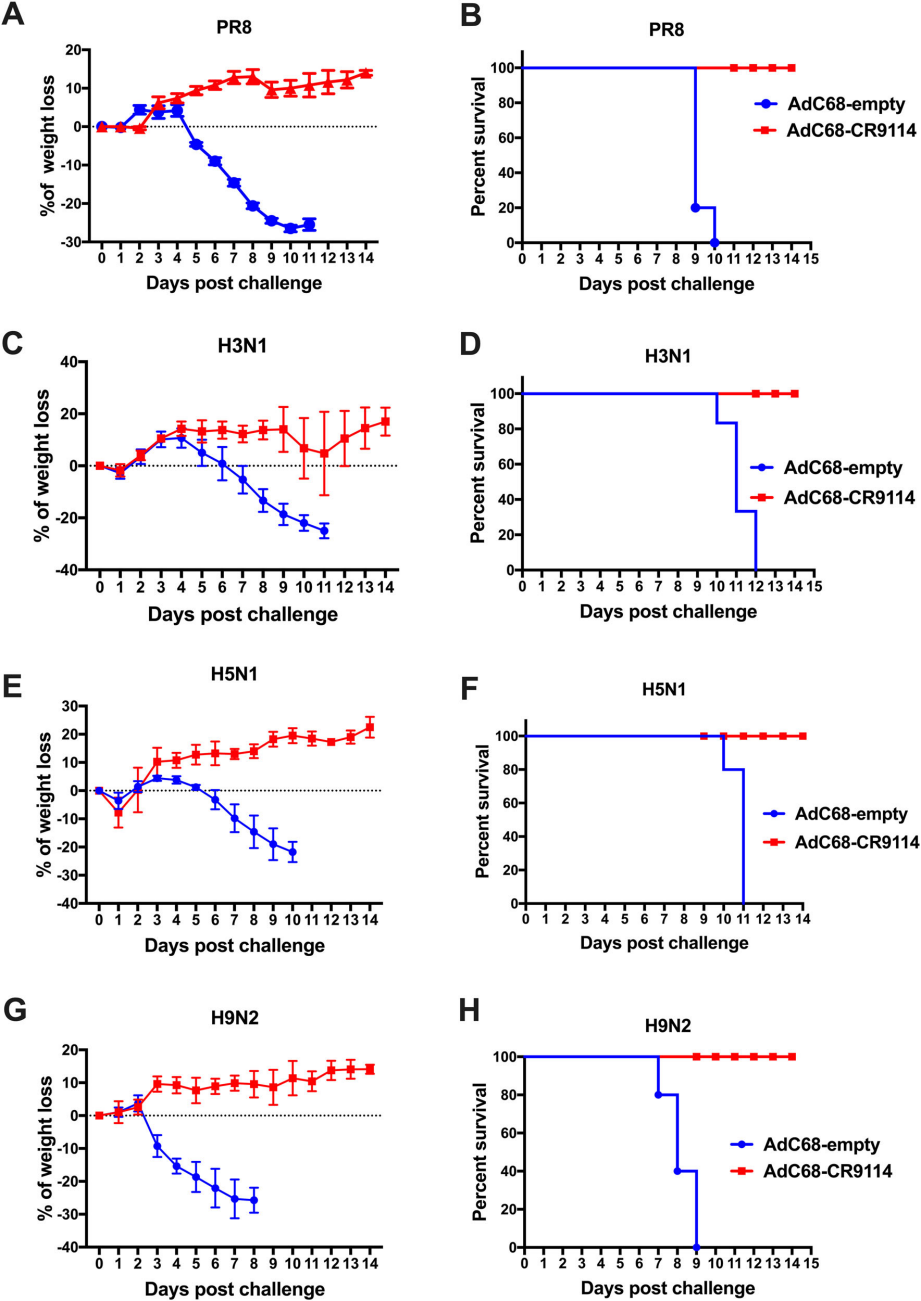
AdC68–CR9114 protected mice from the different subtypes of influenza infection. Mice were intravenously administered 5 × 10^10^ vp AdC68–CR9114 or 5 × 10^10^ vp AdC68–empty, and 24 h post administration, they were challenged with 10LD50 of the different subtypes of influenza virus, PR8 (A/Puerto Rico/8/34), H3N1 (A/Archi/1/1968), H5N1 (A/environment/Hunan/6-69/2008), and H9N2 (A/chicken/Jiangsu/7/2002). Body weight and survival rate were monitored for 14 days. **a**, **c**, **e**, **g** Body weight loss after challenge with PR8, H3N1, H5N1, and H9N2, respectively. Data are expressed as mean ± SD. **b**, **d**, **f**, **h** Survival rate after challenge with PR8, H3N1, H5N1, and H9N2, respectively. Five mice in each group.

To evaluate the potential of AdC68–CR9114 as a universal influenza vaccine for prophylactic use, we conducted a series of challenge studies with different subtypes of the influenza A viruses. Mice were intravenously injected with 5 × 10^10^ vp AdC68–CR9114 or 5 × 10^10^ vp AdC68–empty and challenged with 10LD50 of the different subtypes of influenza virus, covering PR8 (A/Puerto Rico/8/34), H3N1 (A/Archi/1/1968), H5N1 (A/environment/Hunan/6-69/2008), and H9N2 (A/chicken/Jiangsu/7/2002) after 24 h. The PR8 virus is the most widely used virus for influenza research, including influenza vaccine development^25^ and host-virus interaction^26^. H9N2 and H5N1 are two avian influenza viruses that belong to group 1, and H3N1 was selected to represent the group 2 virus. Body weight and the survival rate were monitored for 14 days. As shown in Fig. 4a–h, AdC68–CR9114 conferred full protection against all influenza viruses used in the study. However, no mice in the AdC68–empty group survived the challenge with any of the influenza virus strain.

### Viral loads and the pathological changes in the lungs after pH1N1 virus challenge

**Fig. 5:**
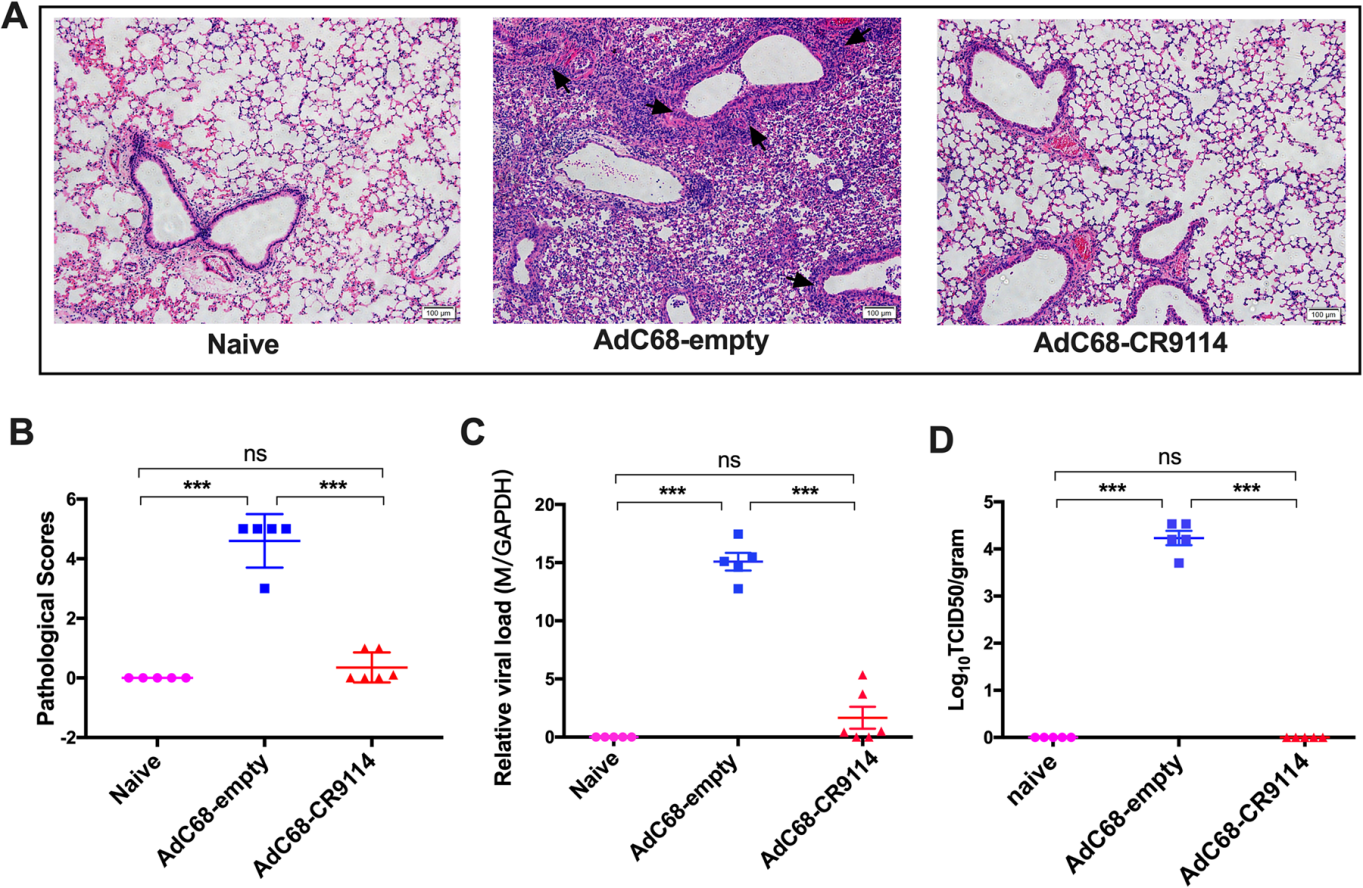
Viral loads and the pathological changes in the lungs after pH1N1 challenge. **a** Five days after pH1N1 challenge, mice were sacrificed, and the lung sections were stained with hematoxylin and eosin. Arrows indicated the perivascular and interstitial infiltration of inflammatory cells and the lung consolidation. **b** Pathological scores of the lungs 5 days after challenge. **c** The viral titer in the lung tissues was determined by quantitative PCR. **d** Lung virus titer was determined by the TCID50 assay. Five mice in each group. ***p < 0.01. ns no significance (determined using one-way ANOVA).

To further evaluate the protective effect of AdC68–CR9114 against the influenza virus, two groups of mice were intravenously administered 5 × 10^10^ vp AdC68–CR9114 or 5 × 10^10^ vp AdC68–empty and challenged with 10LD50 pH1N1 virus after 24 h. All mice were killed on the fifth day post challenge. Thereafter, sections of the lung tissues were fixed and stained with hematoxylin and eosin. Histological analysis of the lungs revealed that mice immunized with AdC68–CR9114 were close to the naive group, with no visible infiltration observed. However, mice in the AdC68–empty group developed severe perivascular and interstitial infiltrates, even necrosis, with an average pathogenic score of 4.6 (Fig. 5a, b). Viral loads were measured via RT-qPCR analysis and a TCID50 assay of the lung homogenates. As shown in Fig. 5c, d, the lung viral titers in the AdC68–CR9114 group were markedly lower than those in the AdC68–empty group.

### Duration of the prophylactic and therapeutic protection induced by AdC68–CR9114

**Fig. 6:**
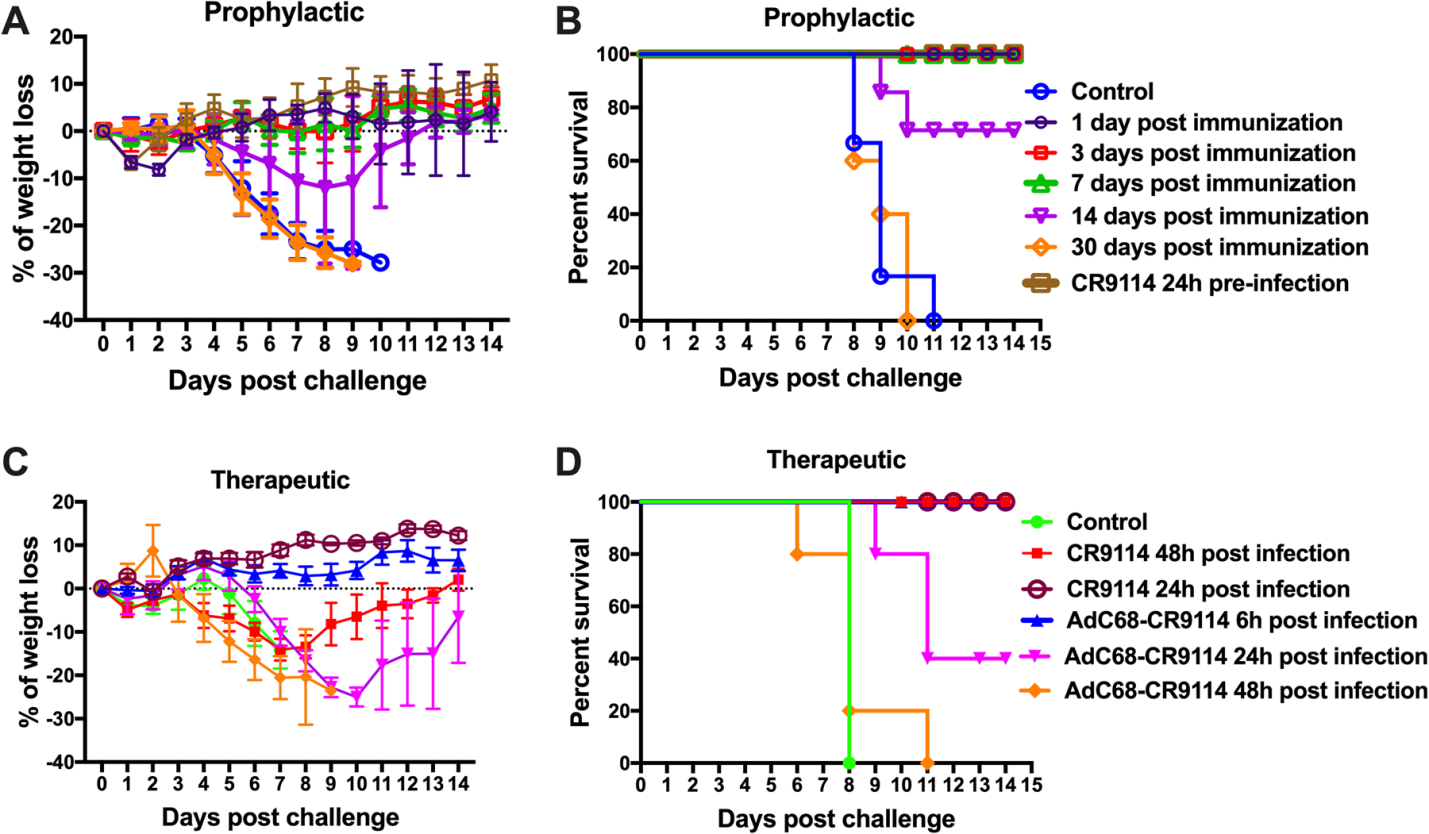
Duration of the prophylactic and therapeutic protection induced by AdC68–CR9114. For the assessment of the prophylactic protection, groups of C57BL/6 mice were treated with intravenous administration of 5 × 10^10^ vp AdC68–CR9114 and challenged with the 10LD50 PR8 virus at different time points. In addition, the mice were intraperitoneally treated with 200 μg of purified CR9114 antibody and then infected with 10LD50 PR8 virus at 24 h later. **a** Body weight change. **b** Survival rate. For the assessment of the therapeutic protection, groups of mice were infected with the 10LD50 PR8 virus and intranasally inoculated with AdC68–CR9114 at different time points or intraperitoneally administered 200 μg of purified CR9114 antibody at 24 or 48 h after infection. **c** Body weight change. **d** Survival rate. Five mice in each group. Data in **a**, **c** are expressed as mean ± SD.

To determine the prophylactic duration of protection, groups of mice were intravenously administered 5 × 10^10^ vp AdC68–CR9114 and challenged with 10LD50 of the PR8 virus on days 1, 3, 7, 14, or 30. In addition, body weight and survival rate were monitored for 14 days. The purified CR9114 antibody and AdC68–empty were used as the positive and negative controls, respectively. As depicted in Fig. 6a, b, AdC68–CR9114 can afford full protection within 7 days post immunization and partial protection on day 14 post immunization. However, protection was not observed on day 30 post immunization.

To further assess the therapeutic timeline effect of AdC68–CR9114, groups of mice were infected with the 10LD50 PR8 virus and intranasally treated with AdC68–CR9114 at different time points. The purified CR9114 antibody inoculated intraperitoneally was used as the positive control. As shown in Fig. 6c, d, AdC68–CR9114 provided full protection from the PR8 virus at 6 h after challenge, partial protection at 24 h, and became invalid after 48 h. Therefore, it is best to treat influenza infection with AdC68–CR9114 within 12 h of its onset. However, the purified CR9114 antibody could confer full protection even at 48 h later (Fig. 6c, d).

## Discussion

Vaccination is one of the most effective strategies against viral infection, including the influenza virus. The most commonly used influenza vaccine is a trivalent inactivated influenza vaccine whose composition is not fixed and can be adjusted according to the WHO’s forecasts. Besides the trivalent inactivated influenza vaccine, live attenuated influenza vaccine^27^ and subunit influenza vaccines are used in the clinic^28^. Sanofi has launched a tetravalent vaccine (VaxigripTetra^TM^) based on the trivalent vaccine administered to individuals ≥3 years old; this vaccine has been approved in Europe^29^. Nevertheless, the efficacy of the influenza vaccine may be significantly attenuated and can be invalid if a large mismatch exists between the vaccine and the new epidemic strain. Antiviral drugs and other treatments are a second intervention to combat influenza infection. Zanamivir and oseltamivir^30^, two neuraminidase inhibitors, have been licensed for clinical use in the treatment of influenza. Adamantane is another anti-influenza drug that targets the M2 protein and blocks the activity of the ion channel, which is essential for viral uncoating following entry into the host cell^31^. However, emergence of drug-resistant influenza virus has been detected^32^, which raises concerns regarding its widespread use. Broadly neutralizing antibodies against influenza would thus be an ideal option in influenza prevention and therapy.

Different strategies for expressing encoded mAbs have been applied in the prevention and control of influenza virus infections. Wilson et al.^33^ revealed that the adeno-associated virus 9 (AAV9) expressing a broadly neutralizing antibody, FI6, (AAV9.FI6) protected mice and ferrets from the heterotypic influenza virus infection when immunized intranasally. In a further study^34^, they found that intranasal immunization with AAV9.FI6 could protect old and immunodeficient mice from the influenza virus. Weiner et al.^35^ used DNA vectors to express two neutralizing antibodies targeting influenza A and B, respectively, and mixed the DNA vectors to protect mice from influenza A and B virus infection. Here, we developed a universal anti-influenza vaccine by using the chimpanzee adenoviral vector, AdC68, expressing CR9114, a broadly neutralizing antibody against influenza virus. AdC68 is a typical chimpanzee adenovirus with no pre-existing immunity in the majority of humans^36^. It has similar immunogenicity to that of AdHu5, and can elicit both humoral and cellular immune responses^37^.

Compared with the AAV vector, the adenoviral vector exerts a relatively short-term expression of the foreign gene of interest^38^. In this study, CR9114 was detected in the serum at 1 day after intravenous injection. Further, its level peaked on day 5 and was reduced to undetectable levels on day 13 post immunization. Hence, AdC68–CR9114 could prophylactically protect mice from influenza challenge for approximately 2 weeks. The influenza virus titer is known to reach its peak at 48 h after infection in human respiratory tracts. Thereafter, it slowly decreases until day 6^39^. Thus, the duration of the neutralizing antibodies expressed by the adenovirus is sufficient to provide protection against influenza virus infection if this inoculation type is administered immediately following the first wave of the influenza outbreak. Based on the therapeutic application, all mice recovered from the influenza infection when intranasally administered AdC68–CR9114 within 6 h after the influenza infection. However, intraperitoneal injection with the purified CR9114 antibody can also be administered to mice suffering from influenza infection up to 48 h. Antiviral drugs are recommended for use within 48 h after infection, with earlier administration leading to better results^40^. Therefore, broadly neutralizing antibodies could be combined with anti-influenza drugs to treat drug-resistant influenza strains. After engineering a multimechanistic influenza B antibody, C12G6, Shen et al.^41^ found that it exhibited an addictive antiviral effect when co-administered with oseltamivir. In future studies, we will explore whether AdC68–CR9114 or purified CR9114 combined with antiviral drugs could synergistically combat influenza infection.

Regarding the antibody expression efficiency, the adenoviral vector is much stronger than the DNA^35^ and AVV vector^33^. The concentration of CR9114 expressed by the adenovirus in the serum reached 11,535 μg/ml at 24 h after intravenous immunization. However, 5 days after mice were administered 300 μg of the DNA plasmid, the serum antibody concentration could only increase to 10 μg/ml^35^. After the AVV9 vector was intranasally administered to mice, the concentrations of the antibody in the nose and lung were 0.5 and 2.0 μg/ml, respectively; however, its concentration in the serum was fairly low (approximately 120 ng/ml). According to the immunization methods employed in our study, 3 or 5 days were required to achieve the peak level of CR9114.

In this study, both intranasal immunization and intravenous injection provided 100% prophylactic protection. However, the concentrations of the neutralizing antibodies in the serum of the intravenously immunized mice were much higher and persisted much longer than in intranasally immunized mice. Thus, intravenous immunization was employed for preventive purposes. However, in the therapeutic experiments, the efficacy of nasal immunization was better than that of intravenous injection. We speculated the reason as the following: in the therapeutic experiment, mice were first infected with the influenza virus and then treated with the adenovirus-encoded antibody 6 h later. If the adenoviruses are administered intravenously, it may result in a toxic and potentially lethal reaction to the mice, and the toxicity of intravenously injected adenoviral vectors may be directly linked to the activation and destruction of Kupffer cells^42^. Although this phenomenon is transient, it may impair the first line of defense of the innate immune system and aggravate the symptoms of influenza infection in mice before the therapeutic antibody getting expressed and exerting its functions. If treated intranasally, adenoviruses were directly delivered to the respiratory tract and the lung; the amount of antibody produced locally might be higher than that of intravenous injection. Importantly, the innate immune system will not be hampered in this type of regimen. Our future efforts are thus focused on further exploiting the advantages of adenoviral vectors and improving the efficacy of this new anti-influenza vaccine.

Compared with the purified CR9114, AdC68–CR9114 was not as effective as purified antibody for therapeutic use in this study. The reason is that the antibody expression by the adenovirus requires time. Generally, antibody expressed by the adenovirus takes at least 24 h to be detected. However, influenza virus replicates quickly once it is seeded in the lung, and reaches its replicative peak 48 h after infection. If the antibody cannot appear in the early stage, it will be less effective. In this study, purified antibodies injected intraperitoneally could directly enter the circulation system to neutralize influenza virus effectively. Thus, the purified CR9114 even given at 48 h post influenza infection could confer full protection in mice, and AdC68–CR9114 was more effective when given within 6 h after influenza virus infection.

Altogether, we developed a universal influenza vaccine (AdC68–CR9114) based on a chimpanzee adenoviral vector that expresses influenza broadly neutralizing mAb. The recombinant adenovirus can express a high level of CR9114 in vitro and in vivo, exert biological functions, and cross-protect mice from different types of influenza virus infection at different time points. Therefore, the findings of this study could provide a landmark strategy for influenza prevention and control, and serve as an example for other infectious diseases.

## Methods

### Cells and viruses

Human embryonic kidney (HEK) 293 cells were bought from ATCC (USA), and Mardin–Darby canine kidney (MDCK) cells were bought from the Cell bank of Shanghai Institutes for Biological Science (Shanghai, China), and they were maintained in complete Dulbecco’s modified Eagle’s medium supplemented with 10% fetal bovine serum (Gibco, USA) and 2% penicillin and streptomycin (New Cell & Molecular Biotech, China). PR8 (A/Puerto Rico/8/34), pH1N1 (pandemic H1N1, A/California/7/2009), H3N1 (A/Archi/1/1968), H5N1 (A/environment/Hunan/6-69/2008), H9N2 (A/chicken/Jiangsu/7/2002), and influenza B (Yamagata lineage, B/phuket/3037/2013) were stored at −80 °C in our laboratory and propagated in chicken embryos for use in all experiments.

### Construction of the recombinant adenovirus, AdC68–CR9114

E1-/E3-deleted pAdC68 vector is a chimpanzee-originated vector that was generated in our lab^43^. We obtained the sequences of the variable gene for the CR9114 light and heavy chain (GenBank: JX213639.1 and GenBank: JX213640.1) from the National Center for Biotechnology Information (NCBI) and combined them with the constant sequence of human IgG1 to obtain the full sequence of CR9114. CR9114 was subjected to codon optimization to improve its expression in human cells by Genewiz Biotech (Suzhou, China). Thereafter, it was cloned into a shuttle vector, pUC57–CASI, under the CASI promoter. CR9114 was then subcloned into an E1-/E3-deleted pAdC68 vector to obtain the recombinant adenoviral plasmid, pAdC68–CR9114. To rescue the recombinant adenovirus, pAdC68–CR9114 was linearized with Pac I and transfected into HEK293 cells using Lipofectamine 2000 (Invitrogen). The virus AdC68–CR9114 was propagated in HEK293 cells and purified by CsCl density gradient ultracentrifugation.

### Antibody purification and identification

HEK293 cells were propagated to 30, 150-mm^2^ dishes and inoculated with AdC68–CR9114. The supernatants were collected, and the CR9114 antibody was purified using a Protein A column (GE Healthcare). The purified antibody was boiled and subjected to SDS-PAGE for Coomassie Blue R-250 staining.

### Western blot

Different quantities of AdC68–CR9114 (10^10^, 10^9^, or 10^8^ virus particles (vp)) were used to infect HEK293 cells in a 6-well plate. AdC68–empty (10^10^ vp) was used as the negative control, while uninfected HEK293 cells were used as blank controls. After 24 h, the cell supernatant was analyzed by reducing and nonreducing Western blotting with a HRP-conjugated anti-human lgG (H&L) (Abcam, China, Cat. No. ab6759-HRP) at a dilution of 1:5000 to detect the expression of the CR9114 antibody. All blots derive from the same experiment and were processed in parallel.

### ELISA

To measure the level of the CR9114 antibody in vitro, different quantities of AdC68–CR9114 (10^10^, 10^9^, or 10^8^ virus particles (vp)) were used to infect HEK293 cells in a 6-well plate. AdC68-gp (10^10^ vp) was used as the negative control, while uninfected HEK293 cells were used as blank controls. The supernatants were harvested at 24 and 48 h after infection. The supernatants and serum samples diluted in suitable proportions were used to determine the concentration of the CR9114 antibody using sandwich ELISA. The anti-human IgG Kappa (70 ng/well) (Southern Biotech, Cat. No. 2060-01) was coated on the ELISA plate at 4 °C overnight and blocked with 5% skim milk (200 μL/well) for 2 h at 37 °C. After blocking, the samples prepared above were added to the plate (100 μL/well) for 2 h at 37 °C. HRP-conjugated mouse anti-human IgG Fc (1:10,000, 100 μL/well) was added to each well and incubated for 1 h at 37 °C. This was followed by the washing steps and then the addition of the 3,3′,5,5′-Tetramethylbenzidine (TMB) substrate (New Cell & Molecular Biotech Co., Ltd., China) to observe the color reaction, which was stopped using a 2 M sulfuric acid (H_2_SO_4_) solution. Absorbance was measured at 450 nm using a microtiter plate reader (Thermo Scientific, Waltham, MA). To calculate the specific content of the expressed CR9114 antibody, a standard curve was generated according to the dosage of the purified CR9114 antibody.

### Animal studies

All animal experiments were approved by the Institutional Animal Care and Use Committee and the Biosafety Committee of Institut Pasteur of Shanghai, while those related to the H5N1 virus were conducted in a biosafety level 3 laboratory following the standard operating protocols approved by the Institutional Biosafety Committee at the Shanghai Public Health Clinical Center, Fudan University. We have complied with all relevant ethical regulations for animal testing and research. Six- to eight-week-old female C57BL/6 mice were purchased from Beijing Vital River Laboratory (Beijing, China) and housed in the Biological Safety Level 2 laboratory of the Institute Pasteur of Shanghai.

For the in vivo expression of the CR9114 antibody, mice were randomly divided into four groups and intranasally immunized with AdC68–CR9114 or AdC68–empty at 5 × 10^10^ vp/mouse in 30 μl of PBS or intravenously administered AdC68–CR9114 or AdC68–empty at 5 × 10^10^ vp/mouse in 200 μl of PBS. All mice were subjected to bleeding every other day.

For the prophylactic protection assay, mice were divided into two groups and primarily immunized with AdC68–CR9114 or AdC68–empty (5 × 10^10^ vp/mouse, in 200 μl of PBS) through intravenous administration via the tail. After 24 h, mice were challenged intranasally with different groups of influenza A and B viruses, such as PR8, pH1N1, H9N2, H5N1, and H3N1 at doses of 10LD50 in 30 μl of PBS. Intranasal immunization was also implemented to evaluate the prophylactic efficacy of AdC68–CR9114. C57BL/6 mice were intranasally treated with 5 × 10^10^ vp of the AdC68–CR9114 virus and challenged with the 10LD50 pH1N1 virus and A/H3N1 virus after 24 h. To explore the prophylactic duration of AdC68–CR9114, mice were intravenously administered AdC68–CR9114 (5 × 10^10^ vp/mouse, in 200 μl of PBS) and challenged with 10LD50 PR8 in 30 μl of PBS after 1, 3, 7, 14, and 30 days. Mice intraperitoneally administered the purified CR9114 antibody (200 μg/mouse) or intravenously injected with the AdC68–empty (5 × 10^10^ vp/mouse, in 200 μl of PBS) served as the positive or negative controls.

For the therapeutic protection assay, mice were challenged with the influenza A virus of PR8 at a dose of 10LD50, and then treated intranasally or intravenously with AdC68–CR9114 or AdC68–empty (5 × 10^10^ vp/mouse, in 200 μl of PBS) at 6, 24, and 48 h, respectively, or treated by an intraperitoneal injection of purified CR9114 antibody (200 μg/mouse, in 1 ml of PBS) at 24 and 48 h, respectively. The body weights and survival rates of all mice were monitored daily for 14 days. The mouse was euthanized when a body weight loss greater than 30% of its pre-challenge weight was observed.

### Viral loads in the lungs

Mice were euthanized on day 5 post challenge to dissect the lung tissues for histology and viral load detection. Briefly, a section of the weighted lung tissues was homogenized in the corresponding volume of DMEM containing 1% BSA and 1% antibiotics to obtain a 10% (w/v) suspension. A series of dilutions of this suspension was added to the monolayers of MDCK cells in 96-well plates, and the cells were cultured in an incubator at 37 °C. After 4 h, the virus titer was detected via a hemagglutination assay. A 50-μL volume of the supernatant from each well was added to a V-type 96-well plate and reacted with 50 μL 1% (v/v) of chicken RBCs (in PBS) for 15 min at room temperature. The virus titer was calculated using the Reed and Muench method^44^. Another section of the lung tissues was homogenized and resuspended in 1 mL of TRIzol reagent to extract total RNA for RT-qPCR analysis in order to measure and quantify the viral loads. Specifically, 1 μg of RNA from each sample was reverse-transcribed to cDNA according to the manufacturer’s protocol for the One-Step RT-PCR Kit (Roche). The viral loads in the lungs were measured via quantitative real-time transcription PCR. The primer pair for the mRNA of the influenza virus was F-5′ AAGACCAATCCTGTCACCTCTGA-3′, R-5′-CAAAGCGTCTACGCTGCAGTCC-3′, while that of the internal reference gene (GAPDH) was F-5′-CAATGTGTCCGTCGTGGATCT-3′, R-5′-GTCCTCAGTGTAGCCCAAGATG-3′. Data were analyzed using the 7900HT System SDS software (Applied Biosystems).

### Histology

The third section of the murine lung tissues was fixed in 4% formaldehyde for 24 h at 4 °C and subjected to hematoxylin and eosin staining as previously described^26^. To assess the pathological changes in the lungs, the score was derived according to the following criteria: (1) no observable pathology, (2) perivascular infiltrates, (3) perivascular and interstitial infiltrates affecting <20% of the lobe section, (4) perivascular and interstitial infiltrates affecting 20–50% of the lobe section, and (5) perivascular and interstitial infiltrates affecting >50% of the lobe section.

### Statistical analysis

All statistical analyses were performed using GraphPad Prism software version 7.0. The difference in the CR9114 antibody titer in vitro and the viral loads in the lungs among the groups were assessed by one-way analysis of variance (ANOVA). *P* values < 0.05 were regarded as statistically significant.

### Reporting summary

Further information on experimental design is available in the Nature Research Reporting Summary linked to this article.

## Supplementary information

Supplementary Information

Reporting Summary

## Data Availability

All data generated or analyzed in this study are available upon request from the authors.
