# Supplementary material for: Adenovirus delivery of encoded monoclonal antibody protects against different types of influenza virus infection
Source: NPJ Vaccines. 2020 Jul 9;5:57. doi: 10.1038/s41541-020-0206-5 (PMC7347565; doi:10.1038/s41541-020-0206-5)

Figure 1A, upper bands (non-reducing)

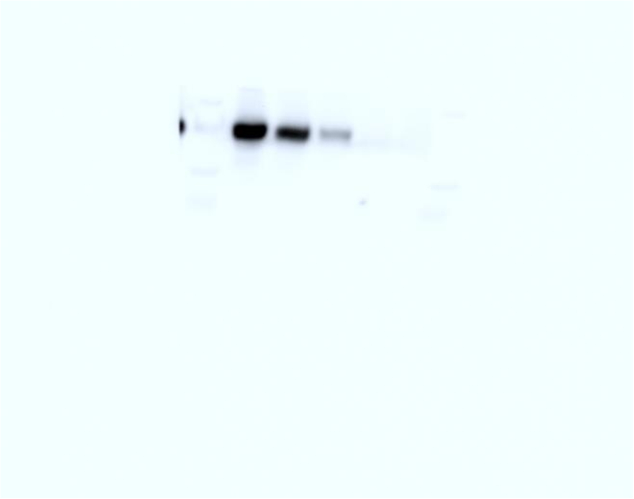

Same gel

Figure 1A, upper bands (non-reducing)
Bright field with Marker

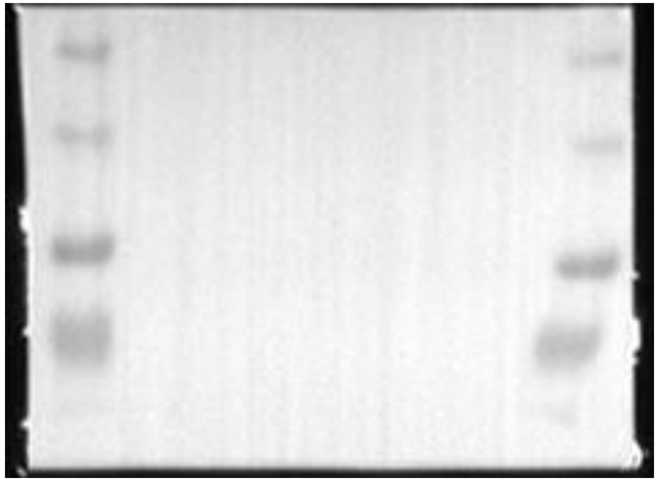

Figure 1A, lower bands (reducing)

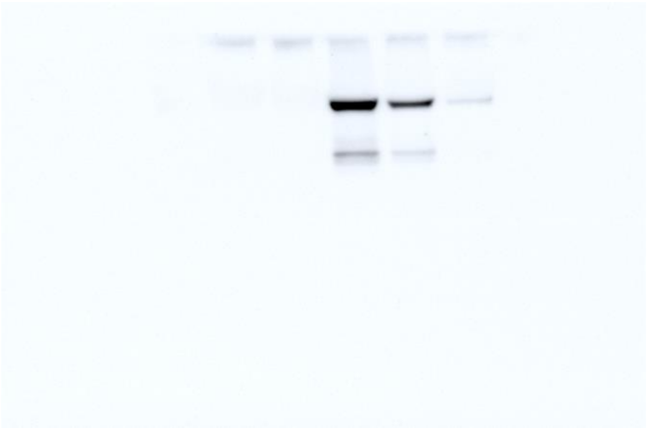

Same gel

Figure 1A, lower bands (reducing)
Bright field with Marker

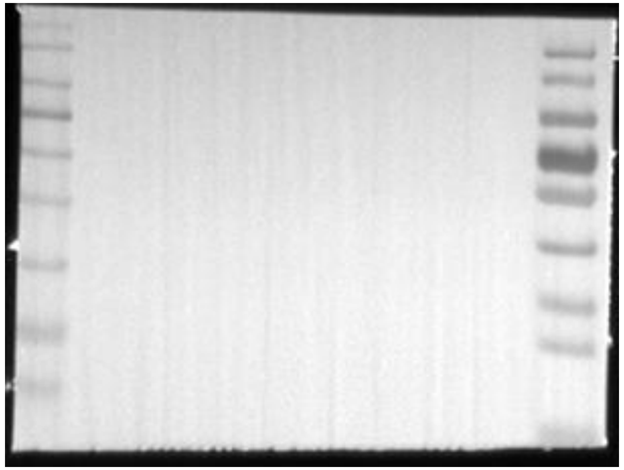

Figure 1B, Coomassie Blue Staining

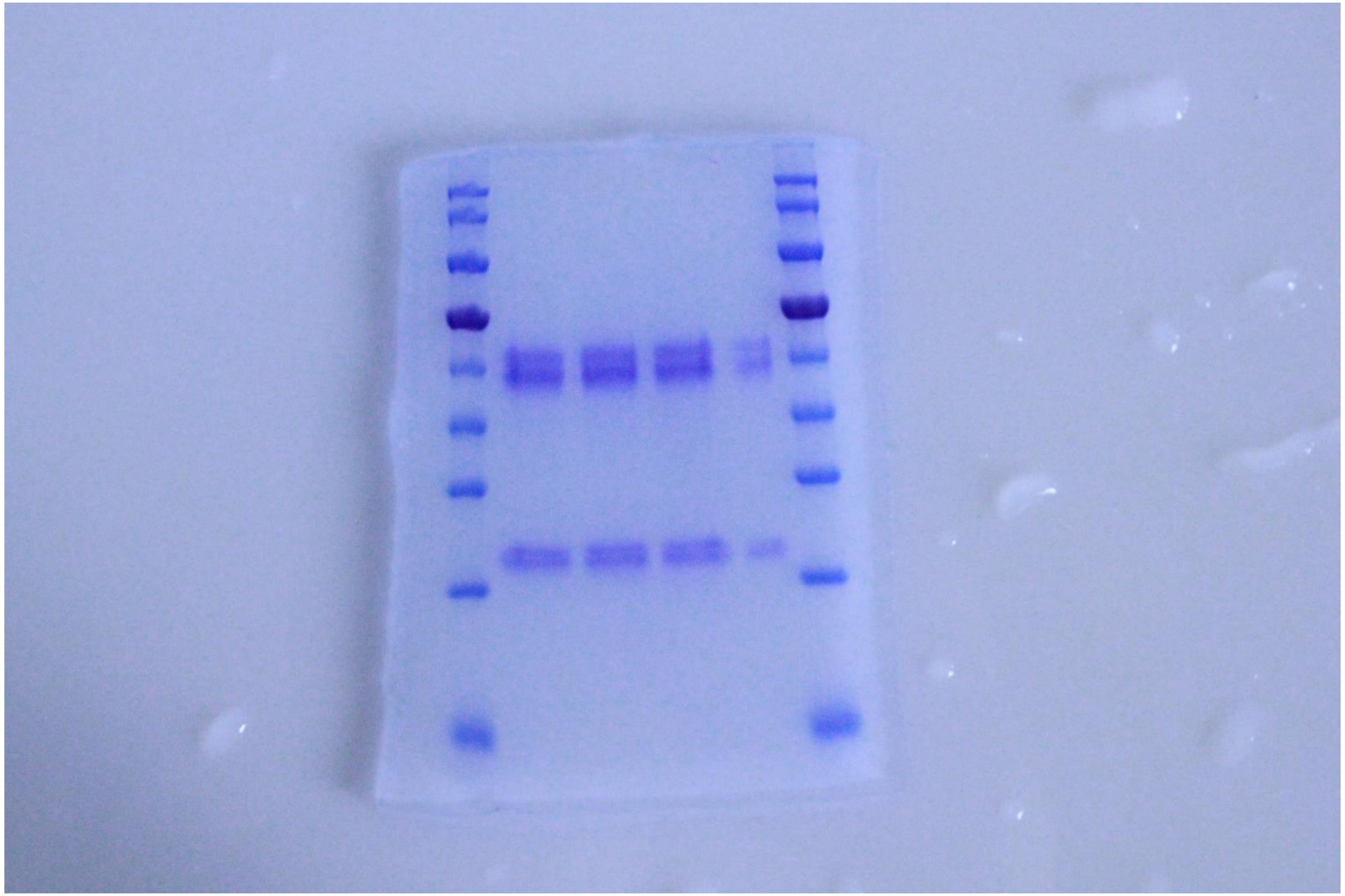

Supplement: Supplementary file 1 — Supplementary Information [file 41541_2020_206_MOESM1_ESM.pdf]
